# Respone to: “Limitations of Retrospective Chart Reviews to Determine Rare Events, and the Unknown Relative Risk of Droperidol”

**DOI:** 10.5811/westjem.2020.9.49870

**Published:** 2020-12-07

**Authors:** Jon B. Cole, Samantha C. Lee, Marc L. Martel, Stephen W. Smith, Michelle H. Biros, James R. Miner

**Affiliations:** * University of Minnesota Medical School, Department of Emergency Medicine, Minneapolis, Minnesota; † Minnesota Poison Control System, Minneapolis, Minnesota; ‡ Hennepin Healthcare, Department of Emergency Medicine, Minneapolis, Minnesota

In reply:

We thank the authors for their interest in our article, and for highlighting some important limitations of our work. 1 We are grateful for the opportunity to address these concerns further.

Regarding the authors’ first concern, indeed we already acknowledge in our limitations section that many of our patients did not receive continuous cardiac monitoring, and asymptomatic events could have been missed. While the clinical importance of asymptomatic self-terminating dysrhythmias is debatable, this question has fortunately been addressed by the DORM II investigators, who prospectively studied patients receiving droperidol for acute behavioral disturbance in multiple Australian emergency departments (ED). All patients in that study were initially treated in a critical care bed and attached to a cardiac monitor. When available, continuous ECG recordings were later analyzed, no patients had dysrhythmias, and while QT prolongation was observed the investigators found it was frequently due to causes other than droperidol. 2 We believe the incidence of such transient asymptomatic dysrhythmias in our study is likely miniscule.

Second, regarding disposition of the patients in our study, while these data were not collected (and are no longer available, as the electronic health record (EHR) from that time has been retired), here we can provide additional clarity. From previously published data we know our mean ED visit length for patients receiving droperidol was approximately seven hours, 3 with the outlier groups being headache (range, 1.5 – 4 hours) 4 and acute agitation (median, 8 hours). 5 It is highly likely patients in the present study had similar visit lengths. This is clinically important, as the recommended observation period in the FDA boxed warning is 2–3 hours. Furthermore, due to droperidol’s short half-life, its clinical effect on the QT interval is likely equally short. In a study of 3,113 patients receiving a mean dose of 4.4 milligrams of droperidol to facilitate endoscopic retrograde cholangiopancreatography in which ECGs were obtained before droperidol and 1–3 hours post-procedure, the authors found that while QT intervals did increase no cardiovascular events attributable to droperidol occurred, and that QT intervals had normalized by the 1–3 hour ECG measurement. 6 Assuming the same is true for ED patients, it is likely that if droperidol-induced torsades des pointes (TdP) occurs, it will do so early in the ED visit. Thus, the risk of missed events in our study is likely low.

We agree with the authors that our study, due to the limitations noted, likely does not determine an exact incidence of droperidol-induced TdP in ED patients. Nevertheless, all studies have an endpoint (ours was the course of usual care for a single ED visit), and our conclusions remain valid within the parameters of our study. We believe, regardless of the precision of our measurement, our data reflect truth in the universe: that droperidol-induced TdP is exceedingly rare, as has been confirmed in other studies both outside 6 and within the ED. 7,8

While the exact incidence of droperidol-induced TdP can be debated, we believe one of the more important findings of our study is that we did find such a case. Drug-induced TdP, in general, is quite rare. When it occurs, it frequently does so in patients with multiple risk factors, 9 which was true with the single case we found. This suggests that it is not the individual medication (ie, droperidol) that requires close monitoring and scrutiny, but rather high-risk patients receiving any QT-prolonging medication. Take, for example, antiemetics, one of the most commonly administered medication classes in the ED. Despite droperidol’s boxed warning, data are clear that the risk of droperidol-induced TdP is quite rare. Ondansetron, a commonly used antiemetic in the ED, has a similarly strongly worded boxed warning for QT prolongation. We are unaware of a study similar to ours that attempts to determine the rate of ondansetron-induced TdP in the ED, despite the fact that in controlled studies, ondansetron causes QT prolongation at similar rates and to a similar degree as droperidol. 10 Most commonly administered antiemetics in the ED are associated with QT prolongation (Table); it remains unclear which of these is safest. Taken in this context, we believe our findings suggest that vigilance and monitoring be focused on high-risk patients for drug-induced TdP, rather than on a specific medication. A vomiting, hypokalemic patient on multiple QT prolonging medications with poor nutritional status should be on a cardiac monitor, regardless of which antiemetic they receive.

We thank the authors for giving us the opportunity to further address our study’s limitations. We join them in calling for rigorous future studies to determine the true incidence of droperidol-induced TdP, and additionally call for similar scrutiny of other commonly administered medications in the ED known to prolong the QT interval.

**Table:** Common antiemetics used in emergency medicine.

Antiemetic	CredibleMeds.org* rating for torsades des pointes	Usual adult dose (IV)	Half-life
Droperidol	Known risk of TdP	0.625 – 2.5 mg	2 hours
Haloperidol	Known risk of TdP	0.5 – 2 mg	14 – 26 hours (IV)
Ondansetron	Known risk of TdP	4 – 8 mg	3 – 6 hours; up to 20 hours with severe hepatic impairment
Promethazine	Possible risk of TdP	12.5 – 25 mg	10 hours (IM) 9 – 16 hours (IV)
Metoclopramide	Conditional risk of TdP	10 – 20 mg	5 – 6 hours
Olanzapine	Conditional risk of TdP	1.25 – 2.5 mg	30 hours (IM; IV half-life unknown)
Prochlorperazine	Not classified	5 – 10 mg	6–10 hours (IV)

* CredibleMeds.org is a non-profit, federally funded, online database of independent information regarding safe medication use. It rates the risk of drug-induced torsades des pointes (TdP) from highest (known risk) to lowest (conditional risk). Definitions for each category of risk are available at www.crediblemeds.org.

*mg*, milligram; *IM*, intramuscular; *IV*, intravenous.
